# Promoting migrant health literacy in adult education, from research to practice

**DOI:** 10.1093/eurpub/ckac131.356

**Published:** 2022-10-25

**Authors:** S Harsch, U Bittlingmayer

**Affiliations:** University of Education Freiburg, Freiburg, Germany

## Abstract

**Background:**

As migration affects health, migrants need context-appropriate health literacy (HL) to maintain and promote health. Possible venues to improve HL are adult education programs, i.e., language courses (LC). However, empirical evidence on these courses and their contribution to HL is scarce. Moreover, general HL recommendations and conceptually developed HL programs often proved unsuitable in practice as they insufficiently incorporate the multiple influences. This paper’s purpose is to show, using key findings, how ethnographic research can contribute to developing appropriate programs and how the transfer from research to practice can succeed.

**Methods:**

The SCURA project conducted extensive ethnographic research on HL in second LC, i.e., 100 h participant observation in 2 classes, 40 teacher interviews, analysis of 24 textbooks, and teacher training. On this basis, we designed programs and drew lessons from comparison with other programs.

**Results:**

The study revealed that health and HL as a situational social practice play a key role in LC e.g., as prerequisites for attendance and learning, part of the syllabus, and an occasion for informal conversations. Multiple concepts of health and HL coexist and influence HL promotion. Besides the setting and course conditions, teachers strongly shape HL activities and students show varying degrees of HL practice. We exposed the complexity and diversity of HL situations, meaning-making processes, strategies to acquire and promote HL, and actual space of action. Then, we developed participatory practicable approaches to promote HL in the educational offer. Compared to other programs, the ethnographic study allowed us to address the major factors, tap into the actual space of action, and increase acceptance, uptake, and sustainability.

**Conclusions:**

Improving HL in existing offers requires a profound understanding of the logic of the field. Ethnographic studies facilitate describing these peculiarities and tailoring interventions.

**Key messages:**

• Ethnographic studies allow understanding the relevant factors, actors, situations, contexts, processes, strategies, the logic within and the scope for action.

• The empirical evidence gained in ethnographic studies enable researchers to develop promising, accepted, and sustainable interventions.

